# Health care consumption among adolescent girls prior to diagnoses of sexual abuse, a case–control study in the Stockholm Region

**DOI:** 10.1007/s00787-019-01445-y

**Published:** 2019-11-29

**Authors:** Gita Rajan, Gunnar Ljunggren, Per Wändell, Lars Wahlström, Carl Göran Svedin, Axel C. Carlsson

**Affiliations:** 1 Division for Family and Community Medicine, Department of Neurobiology, Care Sciences and Society, Karolinska Institutet, Alfred Nobels Allé 12, 141 83 Huddinge, Sweden; 2 Academic Primary Health Care Centre, Stockholm Region, Stockholm, Sweden; 3 Public Health Care Services Committee Administration, Stockholm Region, Box 6909, 102 39 Stockholm, Sweden; 4 Centre for Psychiatry Research, Karolinska Institutet, Stockholm, Sweden; 5 Barnafrid, Child and Adolescent Psychiatry, Department of Clinical and Experimental Medicine, Faculty of Medicine, Linköping University, 581 83 Linköping, Sweden

**Keywords:** Sexual abuse, Administrative databases, Comorbidity, Medication, Epidemiology

## Abstract

Victims of sexual abuse have more co-morbidities than other persons in the same age and the most affected group are adolescent girls. Little is known about how this is reflected in health care consumption patterns prior to the registered diagnosis. The aim of this investigation was to study health care consumption patterns among girls, 12–17 years old, 1 and 2 years prior to their diagnoses of sexual abuse. Through the Stockholm Region administrative database (VAL), data of co-morbidities, number of health care visits, and prescribed drugs were collected for cases (girls age 12–17 with diagnoses of sexual abuse, *n* = 519) and controls matched for age and socio-economic status (*n* = 4920) between 2011–2018. Health care consumption and co-morbidities were significantly higher for the cases compared to controls, with a rise 1 year before the diagnoses: the total number of health care visits (including no shows) 1 year prior to the first recording of the diagnosis was 20.4 (18.1–22.7) for the cases and 6.2 (5.8–6.6) for the controls. The most frequent visits 1 year prior to the diagnosis were to outdoor clinics, with a mean value of 19.1 (16.9–21.3) visits for the cases and 5.7 (5.3–6.1) for the controls, followed by psychiatric clinics with a mean value of 12.7 (10.6–14.8) visits for the cases and 2.0 (1.7–2.3) visits for the controls. The least visited health care clinic 1 year prior to the diagnosis was the emergency ward with a mean value of 1.3 (1.1–1.5) visits for the cases and 0.5 (0.4–0.5) visits for the controls. The most common psychiatric co-morbidities registered among the cases during the first year before the diagnosis of sexual abuse were stress, suicide attempt, and psychosis. Neuroleptics, sleeping pills, antidepressants, and tranquilizers were more frequently dispensed in cases than in controls. Similar patterns were found 2 years prior to the diagnosis. We encourage clinicians to actively ask for exposure of sexual abuse in girls with high health care consumption, making early detection and treatment of sexual abuse available as soon as possible.

## Introduction

In a meta-analysis of 331 international studies it was shown an overall estimated prevalence of self-reported child sexual abuse to be 12.7% (girls 18.0% and boys 7.6%) [31]. International survey and interview studies have shown that the risk of mental illness and substance abuse, as well as physical illness and somatic pain, is more common after interpersonal traumas among both females and males, especially when they have occurred during childhood [1–4, 7–9, 11, 13–15, 19, 22, 26, 27]. Sexual abuse proved to be one of the most common interpersonal traumas during childhood in the Adverse Childhood Experiences (ACE) Study, where 17,337 Americans responded to questions about their childhood experiences [2–4, 7–9, 11–15, 22]. As far as we know, the theoretical and empirical understanding of this topic is mainly based on qualitative and self-report studies based on questionnaires on disclosure, as well as on prevalence. However, in 2017 we presented a register-based study including over two million persons in Stockholm replicating prior results, and also quantifying the results with real numbers and their relative magnitude in comparison with the general population in the Stockholm Region, Sweden. In this study, adolescent girls represented more than half of all cases, and the odds for stress-related diagnoses and depression were tenfolds higher among these girls than in those without a diagnosis of sexual abuse [28]. A limitation to this study was that the co-morbidities and the registered diagnoses of sexual abuse were collected in the same time frame.

In 2018 Fredlund et al. investigated sex as self-injury (SASI), and motives for selling sex among adolescents in Sweden—both behaviors associated with elevated risk for sexual abuse among adolescents [17, 18]. Both SASI and selling sex were associated with poor mental health, in combination with and without a history of child sexual abuse (CSA) [17, 18]. The aim of this study was to study health care consumption patterns among all girls 12–17 years old in Stockholm Region prior to their first recorded diagnoses of sexual abuse and compare to girls without a diagnosis of sexual abuse. Based on previous studies on delayed disclosure and risks of sexual abuse in those with mental health problems, we hypothesize that healthcare consumption is higher already prior to the first diagnoses of sexual abuse.

## Methods

Stockholm Region has today over 2.2 million inhabitants, representing more than one-fifth of Sweden’s entire population. The region includes the capital city of Stockholm and several other cities and towns, as well as large rural areas and a sparsely-populated archipelago. The Stockholm Region is responsible for financing primary and secondary health care, mainly through taxes. With the exception of very few private clinics that operate without subsidies in Stockholm, all consultations and diagnoses are recorded and stored in a central regional database, the Stockholm Regional Health Care Data Warehouse (VAL). The link to VAL makes it possible to perform prevalence and incidence studies for different diagnoses for all residents (and temporary visitors). These databases compile and store data on health care utilization from primary care, specialist open care, hospital inpatient care, and data on collected prescribed medications (23) [6]. As an indication for its accuracy and validity, VAL is used by the Council for updating the National Patient Register kept by the Swedish National Board of Health and Welfare (NBHW) as well as the annual benchmarking reports of the NBHW and the Swedish Association of Local Authorities and Regions (SKL) [23]. Since 1997, diagnoses have been coded according to WHO’s International Classification of Diseases, 10th edition (ICD-10).

### Study population

The studied cohort in the present study was defined as all living girls, age 12–17, who resided in Stockholm Region at some point between 1 January 2011 and 31 December 2018. Data on all health care consultations in primary care and specialized open care during 2011–2018 were extracted from VAL. Girls with a diagnosis of sexual abuse were used as cases. Approximately ten controls per case, matched for age and socio-economic status without recorded diagnosis of sexual abuse were used to compare their consumption of health care. Each control was only enrolled once, even if she matched more than one of the cases. Therefore, the number of controls (*n* = 4920) is less than ten times the number of cases (*n* = 519).

### Socio-demography

We used the Mosaic tool as classification of neighborhood socio-economic status into three levels, i.e., high, middle or low. Mosaic is a tool developed by the marketing company Experian, to classify consumers to make sale activities more effective. The Mosaic system makes it possible to achieve a nuanced classification of socio-economic status. It uses a multivariate modelling utilizing over 400 variables to group postcodes into different types and aggregated broader groups. It uses data from 29 different countries, and has been shown to be useful also for classification of cohorts in epidemiologic research [10, 29].

### Design

This was a case–control study, comparing girls, age 12–17, with a diagnosis of sexual abuse with controls without such a diagnosis. Diagnoses were registered at discharge from hospital or after a consultation 1 year (1–365 days) referred to as year 1, and during 1 year prior to 1 year before (366–730 days), referred to as year 2, before the first diagnosis of sexual abuse was recorded. Only outpatient care data were used. The following ICD codes were used to define the individuals diagnosed with sexual abuse: consultation and observation after a reported rape Z044; sexual abuse by person without weapon Y05 Child sexual abuse, by parent; Y07.8C Child sexual abuse by other than parent; Y07.1C; sexual abuse T74.2.

#### Relevant co-morbid diagnoses were chosen from an earlier study on the same population (18)

Reactions of stress F43; anxiety disorders F40, F41; psychotic disorders F20, F23, F25, F28 and F29; bipolar disorders F30 and F31; alcohol F10, other substance abuse F11–F19; depression F32, F33; pain from stomach, head, muscles and joints R10, R51, R52, G44 and M79; ADHD F90; autism spectrum disorder F84; borderline personality disorder F60.3; eating disorder F50 and self-harm behavior X60-84. ATC codes were selected to mirror sleep disturbances and psychiatric ails: tranquilizers (R06AD02, N05BB), neuroleptics (N05A), propiomazine (N05CM06), antidepressant drugs (N06A), melatonin (N05CH01), hypnotics (N05CF), stimulants (N06BA), and benzodiazepines (N05CD, N05BA).

### Ethics

All data were anonymized and none of the individuals could be identified. Management and analysis based on VAL is part of a continuous quality control of health care utilization in Stockholm Region. Ethical approval has been obtained from the Regional ethical review board in Stockholm to study diseases and their co-morbidities with these data (permits: 2013/2196–31/2, 2016/638–32).

### Statistical methods

Mean values and 95% confidence intervals (CI) were estimated to compare number of health care visits to all professionals between cases and controls. Conditional logistic regression was used to calculate odds ratios (OR) with 95% confidence intervals (CI), to compare the odds of concomitant disorders and prescribed drugs between the cases and the controls. Due to the matching of age and socio-economic status, we did not adjust our data further. Statistical analysis and data management were performed using SAS software, version 9.4 (SAS Institute Inc., Cary, NC).

## Results

The number of health care visits, planned and completed, in total and in different clinics, is presented in Table 1. The total number of visits (including no shows), are higher for the cases than for the controls already 2 years before the first diagnosis of sexual abuse is recorded. There is an increase 1 year before the first diagnosis of sexual abuse, to a mean of 20.1 (17.9–22.3) visits for the cases, and 5.4 (5.1–5.7) visits for the controls. The most frequently used visit to health care year 1 was visits to outpatient clinics, 18.9 (16.7–21.0) for cases and 5.0 (4.7–5.3) for the controls. The least common type of visit year 1 was to the emergency ward, 1.3 (1.1–1.5) and 0.4 (0.4–0.4) for cases and controls, respectively.

**Table 1:** Number of health care visits in adolescent girls with a diagnosis of sexual abuse and their matched controls 1 (1–365 days) and 2 years (366–730 days) prior to the first diagnosis of sexual abuse (2011–2018)

Clinics visited	Cases 1 year prior to diagnosis *n* = 519 (CI 95%)	Controls 1 year prior to diagnosis *n* = 4920 (CI 95%)	Cases 2 years prior to diagnosis *n* = 519 (CI 95%)	Controls 2 years prior to diagnosis *n* = 4920 (CI 95%)
All clinics visits	20.12 (17.91–22.34)	5.39 (5.09–5.69)	10.71 (9.19–12.23)	4.43 (4.14–4.71)
Emergency clinic visits	1.28 (1.08–1.48)	0.40 (0.37–0.43)	0.83 (0.68–0.98)	0.38 (0.35–0.40)
Outdoor clinic visits	18.85 (16.72–20.97)	4.99 (4.70–5.28)	9.89 (8.44–11.33)	4.05 (3.78–4.33)
Psychiatry clinic	12.42 (10.36–14.49)	1.74 (1.52–1.96)	5.69 (4.38–7.00)	1.24 (1.05–1.43)
Primary health care	3.64 (3.16–4.10)	2.22 (2.07–2.37)	2.99 (2.64–3.34)	2.06 (1.92–2.20)
Other specialist clinic	2.04 (1.87–2.21)	0.49 (0.45–0.54)	0.60 (0.45–0.74)	0.38 (0.35–0.42)
Pediatric clinic	2.02 (1.73–2.31)	0.94 (0.87–1.00)	1.43 (1.19–1.68)	0.75 (0.69–0.81)
“No show” at planned visits	1.41 (1.16–1.66)	0.29 (0.25–0.32)	0.75 (0.58–0.92)	0.22 (0.18–0.25)

The clinic recording most of the diagnosis of sexual abuse in this investigation, was the specialist emergency clinic for raped patients, at Södersjukhuset, Stockholm. The clinic only accepts patients who have been exposed to rapes or rape attempts, during the last 4 weeks. The clinic recorded 64.5% of all the first diagnosis of sexual abuse in the investigation. The psychiatric clinics recorded 23.1% of the first diagnosis and the gynecology clinics recorded 8.7% of the first diagnosis of sexual abuse in the investigation. Least first diagnosis was recorded by child emergency ward (2.1%) other clinics (1.2%), and by primary health (0.4%) (data not shown in tables).

Numbers of visits to different health care professional groups are shown in Table 2. The cases made more visits to all health care professionals than the controls. There is an increase year 1, where the cases have more visits to all health care professional groups where the least difference was found among visits to physicians.

**Table 2:** Numbers of visits to different health care professional groups for cases and controls 1 (1–365 days) and 2 years (366–730 days) prior to the first diagnosis of sexual abuse (2011–2018)

Professionals responsible for the visit	Cases 1 year prior to diagnosis *n* = 519 (CI 95%)	Controls 1 year prior to diagnosis *n* = 4920 (CI 95%)	Cases 2 years prior to diagnosis *n* = 519 (CI 95%)	Controls 2 years prior to diagnosis *n* = 4920 (CI 95%)
Psychologist, therapist, social worker	8.87 (7.37–10.36)	1.34 (1.18–1.5)	3.83 (2.93–4.74)	0.88 (0.75–1.01)
Physician	6.35 (5.78–6.92)	2.34 (2.25–2.44)	3.98 (3.39–4.57)	2.15 (2.05–2.25)
Other	4.91 (4.19–5.63)	1.71 (1.56–1.86)	2.90 (2.43–3.37)	1.4 (1.25–1.55)

The odds ratios (ORs) of concomitant disorders for cases and the controls are presented in Table 3. The cases showed significantly higher ORs for concomitant disorders than the controls already year 2. The ORs for all diagnoses except for eating disorder, increased for the cases 1 year before the first diagnosis of sexual abuse.

**Table 3:** Odds ratios for relevant co-morbidities in sexually abused girls compared to non-abused controls 1 (1–365 days) and 2 years (366–730 days) prior to the first recorded diagnosis of sexual abuse (2011–2018)

Diagnoses	OR 1 year prior to diagnosis *n* = 519 (CI 95%)	OR 2 years prior to diagnosis *n* = 519 (CI 95%)
Psychosis (F20, F23, F25, F28, F29)	19.03 (1.72–210.21)	3.17 (0.33–30.49)
Suicide attempt (× 6)	15.51 (7.89–30.51)	5.91 (2.44–14.33)
Stress (F43)	12.37 (7.73–19.79)	7.55 (3.73–15.27)
All substance abuse (F10–F19)	6.29 (3.70–10.70)	3.99 (2.03–7.88)
Alcohol abuse (F10)	6.07 (3.16–11.65)	3.05 (1.30–7.17)
Depression (F32, F33)	5.8 (3.87–8.69)	2.96 (1.54–5.70)
Anxiety (F 40, F41)	5.16 (3.70–7.18)	4.97 (3.18–7.78)
Autism (F.84)	4.25 (2.49–7.24)	3.13 (1.62–6.03)
ADHD (F90)	3.1 (2.22–4.46)	3.02 (2.01–4.53)
Pain (R10, R51, R52, G44, M79)	1.80 (1.43–2.27)	1.53 (1.19–1.96)
Eating disorders (F50)	1.70 (0.65–4.42)	2.65 (0.98–7.17)
Bipolar disorder (F30, F31)	1.59 (0.19–13.17)	> 0.001
Borderline (F60)	−	−

The ORs of dispensed drugs between the cases and the controls are presented in Table 4. The cases had higher ORs for dispensed drugs in all categories analyzed, already 2 years prior to the first diagnosis of sexual abuse.

**Table 4:** Odds ratios for collected prescriptions of relevant pharmacotherapies in sexually abused girls, 1 (1–365 days) and 2 years (366–730 days) prior to the first registered diagnosis of sexual abuse

Pharmaceutical drug type	Odds ratios 1 year prior to diagnosis *n* = 519 (CI 95%)	Odds ratios 2 years prior to diagnosis *n* = 519 (CI 95%)
Tranquilizers (R06AD02, N05BB)	8.91 (6.55–12.14)	5.14 (3.23–8.19)
NRLP (N05A)	8.00 (3.92–16.32)	4.1 (1.57–10.72
Propiomazine (N05CM06)	7.42 (3.58–15.36)	3.68 (1.31–10.35)
Antidepressant drugs (N06A)	7.17 (5.36–9.60)	4.40 (2.94–6.58)
Melatonin (N05CH01)	4.62 (3.35–6.37)	2.58 (1.60–4.20)
Hypnotics (N05CF)	3.80 (0.74–19.66)	−
Stimulants (N06BA)	3.36 (2.40–4.71)	3.63 (2.46–5.34)
Benzodiazepines (N05CD, N05BA)	3.02 (1.20–7.59)	1.19 (0.27–5.17)

## Discussion

The main findings of this case control study were that adolescent girls with a diagnosis of sexual abuse had a significantly higher health care consumption than their controls, matched for socio-economic status and age. This was true already 2 years before the first diagnosis of sexual abuse, with a further increase 1 year before the first recorded diagnosis. The health care visits were mainly to outpatient clinics, but the majority of the first diagnoses of sexual abuse was registered at the emergency clinic for raped patients.

The health care consumption patterns indicate a high burden of mental health problems, demonstrated by high numbers of health care visits, by visits to mental health care professionals, by serious psychiatric diagnosis such as psychosis, suicide attempts, stress and sleep disorders, and by medications thereof. The results also indicate that these health care problems were not temporary, but could be seen already 2 years prior to the first diagnosis of sexual abuse, and that they increased as the recording of the first diagnosis of sexual abuse came closer. The results also indicate that 64.5% of the girls in the investigation got their first diagnosis of sexual abuse recorded due to penetrative abuses or attempts.

### Comparison with other studies

Previous research on adverse childhood experiences (ACEs), among which sexual abuse is the most common for girls, has proven ACEs to be a strong risk factor for mental health problems, suicide risk and re-victimization [5, 16, 18, 32]. Qualitative research on disclosure of childhood sexual abuse (CSA) shows that significant delay of disclosure, often for several years, is common [20, 21, 24, 30]. It also shows that disclosure is a process over time, and that it is not likely to occur spontaneously without a safe context [24, 30]. Another study showed that 26.0% of the female patients (*n* = 298) in a study sample from the specialized emergency ward for raped patients at Södersjukhuset in Stockholm had experiences of CSA prior to the sexual abuse that resulted in the visit to the clinic [25].

The findings in this study support the association between CSA, mental health problems and re-victimization shown in the studies above. Mental health problems due to not yet disclosed CSA could explain both the high health care consumption, co-morbidity, and use of medication prior to the first diagnosis of sexual abuse. Delayed disclosure of sexual abuse may also explain why not all diagnoses were registered at the specialized emergency ward for raped patients at Södersjukhuset in Stockholm. Instead the first diagnosis was often (23.1%) recorded within child psychiatric care after several outdoor visits supporting earlier findings that disclosure is a process over time [24, 30]. The high risk of re-victimization after CSA in combination with delayed disclosure, also makes it plausible that some of the cases with their first diagnosis of sexual abuse registered at Södersjukhuset in Stockholm had experiences of CSA prior to the sexual abuse that resulted in the visit to the clinic. This assumption is also supported by research that has shown a 25% higher amount of reports on sexual assaults to the police, than diagnoses of sexual abuse recorded within the health care system, for the same population during the same period of time [28].

Yet other research mentioned above, has proven ACEs and mental health problems to be risk factors for both sexual risk-taking behaviors and sex as self-injury, behaviors associated with an increasing risk for sexual abuse [5, 16, 18, 32]. Mental health problems could therefore per se explain the high health care consumption, including psychiatric diagnoses and medication, prior to the first diagnosis of sexual abuse. In both cases, the alarming high number of first diagnoses of sexual abuse recorded at the emergency award for raped patients, indicate that the health care consumption patterns in this study are important risk factors for future sexual abuse, regardless of being the first sexual abuse or a situation of re-victimization.

## Clinical implications

The results of this study strongly indicate that adolescent girls, should be questioned of the occurrence of ACEs when having diagnoses, symptoms of, or need of medication for psychosis, ADHD, suicide attempts, stress, anxiety, depression or sleep disorders. Considering difficulties associated with disclosure of CSA, we encourage clinicians to always pose direct questions about CSA, when faced with adolescent girls with these symptoms. The results, however, also highlight the importance for health care clinics encountering patients with above-mentioned diagnoses, to be well-educated and trained in how to talk to, and ask adolescents about ACEs and CSA in a way that offers time for follow-up, and does not intimidate or re-traumatize. When needed, it is also important to be able offer treatment of symptoms in the aftermaths of interpersonal traumas in general, and sexual abuse in particular, as well as effective sexual abuse preventive programs.

### Strengths and limitations

As far as we know, this is the largest investigation of health care consumption prior to diagnoses of sexual abuse, among girls. The well-defined inclusion criteria and the broad data from all forms of care, and from collected and prescribed drugs, are strengths. Another strength is that the diagnostic data were based on clinical assessments and not self-reported. The present study was observational and we cannot draw firm conclusions on causality.

### Conclusion

High health care consumption is common among girls already 2 years prior to a diagnosis of sexual abuse. Diagnoses of in particular psychosis, ADHD, suicide attempts, stress, anxiety, depression or sleep disorders, as well as the need for neuroleptics, hypnotics, and tranquilizers among adolescent girls is also common already 2 years prior to a first diagnosis of sexual abuse. Clinicians should be aware of these associations, and well-educated and trained nurses, physicians or therapists should ask actively for ACEs in general and sexual abuse in particular when these patterns occur. The education and training are necessary to minimize the risks for drop-outs and re-victimization and also to understand the importance to offer time for follow-up. This would facilitate early intervention and treatment if sexual abuse has occurred, and to prevent future sexual abuse from occurring. Qualitative interview studies as well as chronological patient file-studies, to increase the understanding of what has preceded the first diagnosis of sexual abuse, would be of great value.

### What this study adds

This study merges with existing research about delayed disclosure of child sexual abuse, where years of suffering before disclosure of sexual abuse has been reported. It also merges with research on associations between sexual abuse and mental health problems, and on ACE and mental health problems as risk factors for sexual risk taking, sex as self-injury and risks for later sexual abuse. In this study the suffering and risks are visualized and quantified through health care consumption patterns. By recognizing these health care consumption patterns and actively questioning regarding sexual abuse and other ACEs, early intervention and treatment can be made possible.
